# Effect of Methyl Jasmonate Treatment on Primary and Secondary Metabolites and Antioxidant Capacity of the Substrate and Hydroponically Grown Chinese Chives

**DOI:** 10.3389/fnut.2022.859035

**Published:** 2022-04-05

**Authors:** Cheng Wang, Jing Zhang, Jian Lv, Jing Li, Yanqiang Gao, Bakpa Emily Patience, Tianhang Niu, Jihua Yu, Jianming Xie

**Affiliations:** College of Horticulture, Gansu Agricultural University, Lanzhou, China

**Keywords:** hydroponic agriculture, methyl jasmonate, antioxidant capacity, plant metabolites, Chinese chive, *Allium tuberosum*, metabolic profile

## Abstract

Hydroponic culture has become a commercial planting model for leafy vegetables, herbs, and other plants with medicinal value. Methyl jasmonate (MeJA) is involved in primary and secondary plant metabolism; moreover, it regulates plant bioactive compounds and enhances the nutritional and medicinal value of plants. We performed targeted metabolomic analysis of the primary and secondary metabolites in substrate-grown and hydroponic Chinese chive leaves sprayed with MeJA (0, 300, 500, and 800 μM). Using ultra-performance liquid chromatography (UPLC), UPLC tandem mass spectrometry, and chemometric tools, and analyzed the antioxidant activity of these plants. We identified the biomarkers of amino acids (serine, proline, lysine, and arginine) and phenolic compounds (4-coumaric acid and protocatechuic acid) using chemometric tools to distinguish between substrate-grown and hydroponic Chinese chives treated with MeJA. MeJA (500 μM) treatment significantly increased the total sugar and amino acid (essential and non-essential amino acids and sulfur-containing amino acids) contents of hydroponically grown Chinese chives. However, the changes in total sugar and amino acid contents in Chinese chive grown in substrates showed the opposite trend. The organic acid content of hydroponically grown Chinese chives treated with MeJA decreased significantly, whereas that of substrate-grown plants treated with 300 μM MeJA increased significantly. Further, MeJA treatment significantly increased the phenolic content of substrate-grown Chinese chives. Treatment with 800 μM MeJA significantly increased the carotenoid content of substrate-grown Chinese chives and the phenolic content of hydroponic Chinese chives. In addition, the 500 μM MeJA treatment significantly increased the antioxidant activity of Chinese chives in both substrate-grown and hydroponic cultures, and promoted the accumulation of nutrients and bioactive substances. This treatment also improved the flavor quality of these plants and their nutritional and medicinal value. Thus, the results suggested that MeJA-treated plants could be used as value-added horticultural products.

## Introduction

*Allium* species are known for their beneficial effects on human health and biological functions. These health benefits are attributed to the high content of bioactive compounds in *Allium* plants (1, 2). The Chinese chives (*A. tuberosum* Rottl. ex. Spreng.) have a long history of cultivation in China (3). As a perennial herbaceous root vegetable, it is one of the most widely used and studied species in the genus *Allium* (4). Chinese chives have a unique pungent garlic-like flavor and are rich in organic sulfur compounds, polysaccharides, phenols, flavonoids, and other bioactive substances. Moreover, the Chinese chives is rich in nutrients and has high medicinal value (5, 6). Chinese chives extract also plays an important role in the treatment of cardiovascular disease, diabetes, and cancer, and has antibacterial, anti-inflammatory, and antioxidant activities (7, 8). In addition, the characteristic flavor of the Chinese chives has received attention from researchers in recent years (9). As it is used for both food and medicine, improving the nutritional quality, bioactive substances, and antioxidant activity of the Chinese chives can help in increasing its potential nutritional and medicinal value. However, compared with the conventionally cultivated Chinese chives, research on the nutritional quality and bioactive substances of hydroponic Chinese chives has not been conducted extensively.

Plants are natural sources of ascorbic acid (10, 11), minerals (12, 13), and metabolites, such as sugar, amino acid, organic acid, phenolic compounds (14), flavonoids (15, 16), non-flavonoids, beta-carotene (17), betalains (18), and carotenoids (19, 20). Methyl jasmonate (MeJA) is an important cellular regulator that regulates plant development and defense responses to biotic and abiotic stresses (21, 22). In addition, MeJA is certified as a safe compound for all food items when used before harvesting (23). Phytohormone induction induces a response to adversity in plants, and is the main method of increasing the content of secondary metabolites in vegetables (24). Jasmonic acid (JA) and MeJA are the most commonly studied and widely used excitons that used to promote the accumulation of secondary metabolites in horticultural products to improve their nutritional quality (25). Exogenous MeJA can alter the levels of various primary metabolites, including plant sugars, organic acids, and amino acids (26). Treatment with MeJA promotes the production of bioactive compounds in broccoli (*Brassica oleracea* L. var. *italica* Plenck) (27). In addition, MeJA treatment promotes the biosynthesis of volatile compounds and the accumulation of secondary metabolites and non-volatile secondary metabolites (28, 29). Exogenous JA promotes the accumulation of β-carotene and the production of aromatic compounds (30). Liu et al. (31) found that MeJA promoted lycopene accumulation in tomato (*Lycopersicon esculentum*) by upregulating the expression of genes related to carotenoid biosynthesis. Shafiq et al. (32) reported that the application of MeJA promoted the accumulation of phenolic compounds, such as anthocyanin 3-galactoside, chlorogenic acid, and flavonols in the pericarp of apples (*Malus pumila* Mill.). Studies in the field of medical science have shown that a human diet rich in natural plant-synthesized polyphenols can reduce the risk of chronic and degenerative diseases, such as cancer. This is mainly because phenolic compounds rich in anthocyanins, flavonoids, and phenolic acids often exhibit strong free radical scavenging activity (33). MeJA treatment in pomegranate (*Punica granatum* L.) (34) and blackberry (*Rubus fruticosus* Pollich) (35) promotes bioactive compounds and enhances their antioxidant activities and properties beneficial for human health.

Despite their growing popularity, the nutritional quality and bioactive substances of Chinese chives have been relatively less studied, especially compared to those of other *Allium* vegetables, such as garlic and onions. To the best of our knowledge, previous studies have not reported the effects of MeJA treatment on primary and secondary metabolites in Chinese chives. In addition, there is a lack of information on the effects of MeJA treatment on primary and secondary metabolites in other *Allium* crops. Compared to soil cultivation, hydroponics can provide more efficient nutrient requirements for the plants (36). In addition, hydroponic cultivation mainly improves the yield and accumulation of functional components (37). We hypothesized that the two cultivation methods, substrate culture and hydroponic culture, could induce differences in the content of primary and secondary metabolites in Chinese chives, while the application of MeJA could promote primary and secondary metabolites in substrate-grown and hydroponic Chinese chives, consequently, increasing their antioxidant activity and medicinal value. The objective of this study was to determine how foliar spraying with MeJA affected the metabolites of substrate-grown and hydroponic Chinese chives. To better understand the metabolic fluxes between primary and secondary metabolites, we analyzed primary metabolites (including amino acids, organic acids, and sugars) and secondary metabolites (including phenolic compounds and carotenoids) in substrate-grown and hydroponic Chinese chives treated with MeJA. Our findings are important for the development of cultivation systems that can enhance the nutritional characteristics of vegetables.

## Materials and Methods

### Plant Material and Experimental Design

**FIGURE 1:**
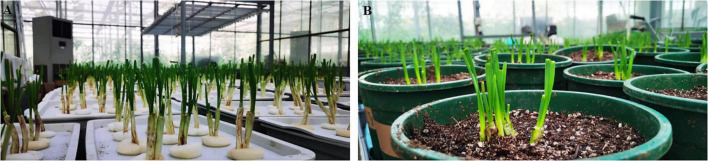
Hydroponic **(A)** and substrate-grown **(B)** Chinese chives grown in a glass solar greenhouse.

Seeds of *A. tuberosum* cv. “Chive God F1” were used as the experimental material. On May 8, 2020, the seedlings were cultivated in Wushan (34°25′–34°57′ N, 104°34′–105°08′ E), China, in the core demonstration area for Chinese chives. On July 20, 2021, we harvested the aboveground leaves and old roots of 1-year-old Chinese chive seedlings, removed the leaves and extra old roots while retaining 2–3 cm of old roots to promote the development of new roots. The plants were then transplanted into hydroponic and substrate cultivation systems in a glass solar greenhouse at Gansu Agricultural University in Lanzhou (36°03′ N, 103°40′ E) (Figure 1). The temperature and relative humidity conditions in the greenhouse were 20 ± 3°C/15 ± 3°C (day/night) and 60–70%, respectively. The nutrient solution for the hydroponic system was prepared according to Wu et al. (38) (Table 1), and the nutrient solution electrical conductivity and pH were 2.13 ms cm^–1^ and 6.15, respectively. Seedlings were fixed with cotton in 16 rectangular hydroponic boxes (0.37 m × 0.25 m × 0.2 m), each having 11 holes. Two seedlings were fixed in each hole; correspondingly, 88 plants were fixed in four boxes per treatment. The nutrient solution was continuously aerated with an air compressor and replaced every 3 d. In the substrate systems, the cultivation substrate, perlite, and vermiculite were provided by Gansu Green Energy Ruiqi Biotechnology Co., Ltd. (Tianzhu, China). The cultivation substrate, which was mainly composed of peat, coconut bran, cow dung, and perlite, was mixed with perlite, and vermiculite in a ratio of 3:1:1 in a 4-L plastic pot, and four seedlings were transplanted in each pot, that is, out of the total 88 pots for four treatments, 22 pots containing 88 seedlings were used per treatment. Each pot of substrate was supplied with 150 mL of the nutrient solution, the composition of which is listed in Table 1, on the first day after transplanting Chinese chive seedlings. In addition, substrate cultivation was watered once or twice per week.

**TABLE 1:** Composition of the nutrient solution for hydroponic.

Compounds	Concentration (mg L^–1^)
Ca (NO_3_)_2_⋅4H_2_O	240
KNO_3_	619
(NH_4_)_2_SO_4_	270
MgSO_4_⋅7H_2_O	248
NaFe-EDTA	30
H_3_PO_4_	0.14
H_3_BO_3_	2.86
MnSO_4_⋅4H_2_O	2.13
ZnSO_4_⋅7H_2_O	0.22
CuSO_4_⋅7H_2_0	0.08
(NH_4_)_6_Mo_7_O_24_⋅7H_2_O	0.02

The experiment was conducted in a completely randomized design with three replicates. The hydroponic Chinese chives were treated with the following exogenous hormones: an aqueous solution of MeJA (containing 0.1% ethanol and 0.1% Tween-20) at three concentrations (300 μM, HM300; 500 μM, HM500; and 800 μM, HM800) and a control solution (0 μM, HCK, containing 0.1% ethanol and 0.1% Tween-20). The substrate-grown Chinese chives were treated with the following exogenous hormones: an aqueous solution of MeJA (containing 0.1% ethanol and 0.1% Tween-20) at three concentrations (300 μM, SM300; 500 μM, SM500; and 800 μM, SM800) and a control solution (0 μM, SCK, containing 0.1% ethanol and 0.1% Tween-20). When the seedlings had grown to approximately 15 cm, 150 mL of the aforementioned aqueous solutions were sprayed on the plant leaves in each treatment group (i.e., total 88 plants) at 7:00–8:00 every morning from August 11, 2021, to August 17, 2021. After 35 d (August 24, 2021), Chinese chives were harvested from all treatment groups. In total, 60 Chinese chive plants were randomly selected for each treatment, with 20 plants in each of three biological replicates. All samples were ground to a powder in liquid nitrogen and stored at −80°C prior to analysis.

### Analysis of Soluble Sugars and Organic Acids by High-Performance Liquid Chromatography

The sugar contents of samples were determined using High-Performance Liquid Chromatography (HPLC) (Agilent series 1100, Agilent Technologies, Santa Clara, CA, United States). The extraction and determination methods were based on Wei et al. (39), with minor modifications. Briefly, frozen Chinese chive powder (fresh weight, 0.5 g) was homogenized with ultrapure water (2.5 mL) and extracted by ultrasonication at 30°C for 60 min, followed by centrifugation at 10,000 × g for 10 min at 4°C. The supernatant (2 mL) was filtered through a 0.22-μm aqueous microporous membrane into a liquid chromatography vial for measurement. The conditions for separating the soluble sugars were as follows: detector, differential refractive index detector (Agilent series 1100, Agilent Technologies); column, LC-NH2 (4.6 × 250 mm); column temperature, 30^°^C; phase, acetonitrile: water (3:1, v: v); flow rate, 1.0 mL min^–1^; and injection volume, 25 μL. The concentrations of sugar compounds were determined based on the areas of their extracted chromatograms using the reference standards of fructose, glucose, and sucrose. Supplementary Figure 1 shows the chromatogram of sugars.

The levels of organic acids were determined using ultra-performance liquid chromatography (UPLC, Waters Corp., Milford, MA, United States). The extraction and determination methods were based on Coelho et al. (40), with minor modifications. Briefly, frozen Chinese chive powder (fresh weight, 1.5 g) was mixed with ultrapure water (7.5 mL) to extract the organic acids. The extract was centrifuged at 4°C and 10,000 × g for 10 min, and the supernatant was aspirated and filtered through a 0.22-μm aqueous phase microporous membrane into a liquid chromatography vial for measurement. The conditions for separating the organic acids were as follows: detector, UV detector; column, Thermo Hypersil COLD AQ (4.6 × 150 mm, 3 μm); column temperature, 30°C; phase, 20 mmol L^–1^ NaH_2_PO_4_ (pH = 2.7); flow rate, 0.5 mL min^–1^; and injection volume, 20 μL. The concentrations of organic acids were determined based on the areas of their extracted chromatograms using the reference standards of oxalic acid, citric acid, and malic acid. Supplementary Figure 2 shows the chromatogram of organic acids.

### Analysis of Amino Acids by Ultra-Performance Liquid Chromatography Tandem Mass Spectrometry

The underivatized amino acids in Chinese chives were quantitatively analyzed by hydrophilic interaction chromatography (HILIC) using liquid chromatography/mass spectrometry (LC/MS) detection (Agilent 1290–6460, LC/MS, Agilent Technologies) (41). In brief, frozen Chinese chive powder (fresh weight, 0.1 g) was extracted with 0.5 M aqueous hydrochloric acid (1 mL). The solution was mixed by vortexing at 8,000 × g for 20 min, sonicating for 20 min in a 25°C water bath, and then centrifuging at 20,000 × g for 20 min. The supernatant (250 μL) was transferred to a liquid chromatographic vial with ^15^N-enriched deuterated internal standard (ISTD), and then diluted to 1 mL with 80% acetonitrile aqueous solution. The HPLC conditions were as follows: column, Agilent Infinity Lab Poroshell 120 HILIC-Z (2.1 × 100 mm, 2.7 μm); phase A, 20 mM ammonium formate (pH = 3) with water at a ratio of 9:1; phase B, 20 mM ammonium formate (pH = 3) with 90% aqueous acetonitrile at a ratio of 9:1; flow rate, 0.5 mL min^–1^; column temperature, 25^°^C; injection volume, 1 μL; total running time, 15 min; gradient time (min), 0, 11.5, and 12; and gradient concentration (%B), 100, 70, and 100. The mass spectrometry conditions were as follows: ionization mode, electrospray ionization in positive ion mode; dryer temperature, 330°C; gas flow rate, 13.0 L min^–1^; atomizer, 35 psi; sheath gas temperature, 390°C; sheath gas velocity, 12 L min^–1^; capillary voltage, 1,500 V; and nozzle voltage, 0 V. Supplementary Figure 3 shows the chromatogram of amino acids. The limit of detection (LOD) and limit of quantification (LOQ) values are listed in Supplementary Table 1.

### Analysis of Phenolic Compounds by High-Performance Liquid Chromatography and Determination of Total Phenol Content

We analyzed polyphenols using an HPLC system (Waters Corp.) equipped with a 1525 pump and a 2998 photodiode array detector. The phenolic compounds were determined according to Liu et al. (42), with minor modifications. Frozen Chinese chive powder (fresh weight, 0.1 g) was added to methanol (2 mL) and placed in a refrigerator at 4°C for 60 min after shaking several times. The solution was centrifuged at 8,000 × g for 10 min at 4^°^C, and the supernatant was filtered through a 0.22-μm filter membrane into a liquid chromatography vial for detection. The conditions for separating the phenolic compounds were as follows: column, Waters Symmetry C18 column (4.6 × 250 mm, 5 μm); mobile phase, methanol and 1% acetic acid for gradient elution; flow rate, 1.1 mL min^–1^; column temperature, 30°C; and injection volume, 10 μL. The phenolic compounds were detected at 240, 280, and 322 nm (Table 2). P-hydroxybenzoic acid, protocatechuic acid, quercetin, chlorogenic acid, rutin, cinnamic acid, 4-coumaric acid, gallic acid, benzoic acid, ferulic acid, erucic acid, caffeic acid, artichoke element, kaempferol, and gentilic acid (Sigma-Aldrich, Burlington, MA, United States) were used as external standards. Supplementary Table 2 provides the retention time, wavelengths of maximum absorption in the visible region (λ_*max*_) and temporary identification of the phenolic components. Supplementary Figure 4 shows the phenolic acid chromatogram. The LOD and LOQ values are listed in Supplementary Table 3. Total phenol content was determined according to the method of Hand et al. (43) with minor modifications. Briefly, a crude extract of phenol was extracted with 50% methanol, mixed with distilled water, and then extracted with Folin-Ciocalteu phenol reagent and 20% sodium carbonate solution. After 30 min in a water at 50°C in the dark, the absorbance was measured at 760 nm using a UV-1780 spectrophotometer (Shimadzu Instruments, Suzhou, China). Total phenol content was calculated using a calibration curve for gallic acid.

**TABLE 2:** Determination of phenolic compounds by HPLC at three wavelengths.

Wavelength	240 nm	280 nm	322 nm
Phenolic compounds	P-hydroxybenzoic acid	Cinnamic acid	Erucic acid
	Protocatechuic acid	4-coumaric acid	Caffeic acid
	Quercetin	Gallic acid	Artichoke element
	Chlorogenic acid	Benzoic acid	Kaempferol
	Rutin	Ferulic acid	Gentilic acid

### Analysis of Carotenoids by High-Performance Liquid Chromatography

Carotenoids were determined according to Li et al. (44), with minor modifications. Frozen Chinese chive powder (fresh weight, 2 g) and butylated hydroxytoluene (0.1 g) were homogenized by grinding with a small volume of liquid nitrogen and transferred to a 50-mL centrifuge tube. This was added to 20% KOH-methanol solution (8 mL), and the mixture was sealed and placed in a constant water bath at 55°C for 30 min. The extract was added to a 1:2 solution of acetone: ethyl acetate, ultrasonicated for 40 min, and centrifuged at 8,000 r min^–1^ at 4^°^C. The supernatant was concentrated by rotary evaporation, and the volume was made up to 10 mL with acetone. For HPLC detection, the solution was filtered into the injection bottle with a 0.22-μm organic membrane. Samples were analyzed using an HPLC system (Analytical HPLC, 1260 Infinity II LC System; Agilent Technologies) coupled to a diode array detector. The conditions for separating the carotenoids were as follows: column, a special Welch Ultimate C30 column (4.6 × 250 mm, 5 μm) for carotenoids; column temperature, 30^°^C; flow rate, 1.0 mL min^–1^; injection volume, 20 μL; phase A, acetonitrile; phase B, water; phase C, methyl tertbutyl ether: methanol (V: V = 1: 1); and phase D, ethyl acetate. The carotenoids were detected at 470, 443, and 286 nm (Table 3) and quantified using the reference standards of α-carotene, monoepoxy zeaxanthin, zeaxanthin, lycopene, violaxanthin, lutein, and β-carotene. Supplementary Figure 5 shows the chromatogram of carotenoids. The LOD and LOQ values are listed (Supplementary Table 4).

**TABLE 3:** Determination of carotenoids by HPLC at three wavelengths.

Wavelength	286 nm	443 nm	470 nm
Carotenoids	Monoepoxy zeaxanthin	Lycopene	α-carotene
			Zeaxanthin
	Violaxanthin		Lutein
			β-carotene

### Antioxidant Capacity of Chinese Chives

The 2, 2′-diphenyl-1-picrylhydrazyl radical (DPPH) assay for scavenging activity and ferric reducing/antioxidant capacity (FRAP) assay and 2,2′-azino-bis (3-ethylbenzothiazoline-6-sulfonic acid) (ABTS) assay for free radical scavenging activity were performed using a commercially available kit (Suzhou Keming, Suzhou, China) according to the manufacturer’s instructions. The sample was homogenized in an ice bath at a 1:10 ratio of sample fresh weight (g): volume of extract (mL), and then centrifuged at 10,000 × g for 10 min at 4°C. The supernatant was kept in ice for testing, and the supernatant and reagents were added to the enzyme plate in turn according to the instructions. The enzyme plates for the DPPH and FRAP assays were reacted for 20 min at 20–23^°^C. The absorbance was measured at 515 and 593 nm for DPPH and FRAP assays, respectively, using a multi-function microplate reader (Spectramax i3, Molecular Devices, Sunnyvale, CA, United States). A multifunctional microplate reader was used for the ABTS assay to detect the absorbance at 734 nm immediately after adding the reagents. Trolox was used to establish the standard curve, and the scavenging activity and reducing ability were expressed in Trolox equivalents (μmol Trolox g^–*l*^ FW).

The oxygen radical absorbance capacity (ORAC) was determined using a kit (Huicheng Biological Technology, Shanghai, China) according to the manufacturer’s instructions. In brief, the sample was homogenized in an ice bath at a 1: 10 ratio of sample fresh weight (g): volume of ethanol (mL), and then centrifuged at 10,000 × g for 10 min at 4°C. The supernatant was placed in ice for further testing. The supernatant (50 μL) and 10 μL mL^–1^ sodium fluorescein (50 μL) was mixed in a black fluorescent enzyme plate and left to react in the dark for 30 min at 37°C. Subsequently, a free radical initiator was added, and the mixture was immediately subjected to fluorescence enzyme kinetic assay using a multi-function microplate reader (Synergy HTX, BioTek Instruments Ltd., Winooski, VT, United States). Readings were recorded every 3 min for 90 min at an excitation wavelength of 485 nm, an emission wavelength of 520 nm, and an incubation temperature of 37°C. The recordings were analyzed using the Trolox calibration curve and the area under the curve (AUC) of the fluorescence decay. The results were expressed as micro-moles of Trolox equivalent per mL. The AUC was calculated as follows:


$$A\,U\,C\,=\,F_{0}/F_{0}+\,F_{1}/F_{0}+\,F_{2}/F_{0}+\ldots \,\ldots +\,F_{n}/F_{0}$$

where F_0_ is the initial fluorescence reading at 0 min, and F_*n*_ (*n* = 31) are the fluorescence readings taken every 3 min up to 90 min.

### Statistical Analysis

All experimental data were analyzed using IBM SPSS Statistics version 21.0 (SPSS Inc., Chicago, IL, United States). The statistical significance of differences in treatment means was evaluated by Duncan’s multiple range test (*p* < 0.05). All data are presented as the mean ± standard error of three biological replicates. The figures were generated using Origin Pro 2021. For multivariate analysis, raw data were normalized by the sum, mean-centered, and divided by the square root of the standard deviation of each variable (Pareto scaling). We also conducted principal component analysis (PCA) and partial least squares discriminant analysis (PLS-DA), and calculated the variables importance in projection (VIP) scores. To evaluate differences between treated and untreated groups, heat maps (with Pearson’s correlation coefficients as a distance measure) were illustrated using the MetaboAnalyst 5.0 server (accessed on 20 October 2021).^1^

## Results and Discussion

### Effect of Methyl Jasmonate Treatments on Sugars and Organic Acids (Primary Metabolites) in Chinese Chive

**FIGURE 2:**
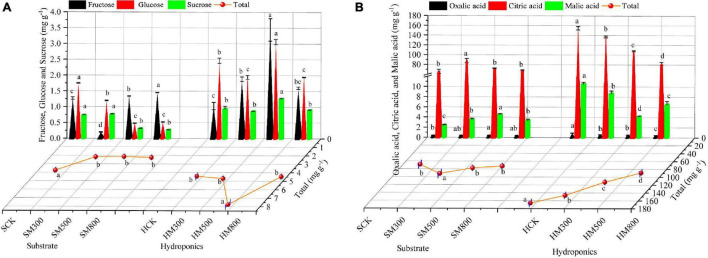
The sugar **(A)** and organic acid **(B)** contents of substrate-grown and hydroponic Chinese chives treated with methyl jasmonate (MeJA). Data represent the mean ± standard error (*n* = 3). Different lowercase letters indicate statistically significant differences according to Duncan’s multiple range tests (*p* < 0.05).

The sugars and organic acid contents of substrate-grown and hydroponic Chinese chives treated with MeJA are shown in Figure 2. The fructose content of substrate-grown Chinese chives increased with the MeJA treatment (500–800 μM), and was significantly higher than that of the SCK group. In contrast, the MeJA treatment significantly reduced the glucose, sucrose, and total sugar contents of substrate-grown Chinese chives (Figure 2A). Tytgat et al. (45) reported that JA treatment reduced the sugar concentration of kale (*Brassica oleracea* L.), which is similar to our results for substrate-grown Chinese chives. However, treatment with 500 μM MeJA significantly increased the fructose, glucose, sucrose, and total sugar contents of hydroponic Chinese chives compared to those in the HCK group. In addition, we found that fructose and glucose constituted the main sugar fraction in hydroponic Chinese chives, accounting for 79–84% of the total sugars. In contrast, fructose constituted the main sugar fraction in substrate-grown Chinese chives, accounting for 10–65% of the total sugars. Liang et al. (46) reported that the MeJA treatment reduced the levels of most sugars in potted radish (*Raphanus sativus* L.), which was contrary to the results of our hydroponically grown plants. This suggested that the cultivation method, which results in high sugar content in hydroponic plants, is an important factor affecting the sugar contents of hydroponic and substrate-grown Chinese chives.

Citric acid is found in many fruits and vegetables, and its proper supplementation can promote appetite and enhance normal metabolism in the body (47). In this study, we found that citric acid constituted the main organic acid fraction in Chinese chives, accounting for 94–96% (substrate-grown) and 92–96% (hydroponic) of the total acid content. This was followed by malic acid, accounting for 3.6–5.9% (substrate-grown) and 3.7–7.3% (hydroponic) of the total acid content (Figure 2B). In addition, treatment with MeJA (300 μM) significantly promoted the citric and total acid contents of the substrate-grown Chinese chives. This is consistent with the findings of Kim et al. (48), who found that MeJA increased the levels of citric and organic acids in potted cabbage (*Brassica oleracea* L.). The MeJA treatment also significantly increased the malic acid content, which accelerates the scavenging of free radicals in human tissues (49). However, organic acid contents in hydroponic Chinese chives decreased significantly with increasing MeJA concentrations (Figure 2B). This indicated that the effects of MeJA on the organic acid content of Chinese chives differed between different cultivation methods. Oxalic acid is an anti-nutritional factor that reduces the effectiveness of calcium (a mineral element) in vegetables. It also combines with minerals in other foods to form oxalates, which are difficult for the body to absorb and may cause kidney and urinary tract stones with a poor long-term diet (50, 51). It is generally accepted that vegetables containing oxalic acid should not be consumed at the same time as foods high in calcium (52). In this study, we found that the oxalic acid content of hydroponically grown Chinese chive leaves (0.88 mg g^–1^) was higher than that of the substrate-grown Chinese chives (0.41 mg g^–1^). However, the oxalic acid content of the MeJA-treated hydroponically grown Chinese chives decreased significantly with increasing MeJA concentration. This offers a potential way to reduce the oxalic acid content of vegetables with high calcium content, thus, avoiding inadequate calcium absorption by the body due to the high oxalic acid content of vegetables.

### Effect of Methyl Jasmonate Treatments on Amino Acids (Primary Metabolites) in Chinese Chive

The levels of 18 amino acids in the substrate-grown and hydroponic Chinese chives treated with MeJA are listed in Table 4. Substrate-grown and hydroponic Chinese chives were rich in lysine (73.66 and 62.50 mg g^–1^), threonine (1.44 and 1.41 mg g^–1^), phenylalanine (1.51 and 1.45 mg g^–1^), tryptophan (0.34 and 0.47 mg g^–1^), leucine (1.76 and 1.65 mg g^–1^) (1.84 and 1.75 mg g^–1^), isoleucine (1.84 and 1.75 mg g^–1^), valine (2.28 and 2.12 mg g^–1^), and methionine (1.76 and 1.14 mg g^–1^). Collectively, these accounted for 55 and 54% of the total amino acid content in substrate-grown and hydroponic Chinese chives, respectively, indicating that these plants were good sources of essential amino acids (EAAs). Among non-essential amino acids (NEAAS), glutamate levels were the highest in substrate-grown and hydroponic Chinese chives (26.02 and 18.72 mg g^–1^, respectively), whereas cysteine levels were the lowest. Amino acids are often involved in various biochemical mechanisms, such as protein synthesis, cell signaling, osmoregulation, and metabolic regulation (53). Tytgat et al. (45) reported that JA treatment reduced the levels of amino acids in kale, and Liang et al. (46) found that MeJA application reduced amino acid content in Brassica. These are similar to our results of substrate-grown Chinese chives, which showed significantly lower levels of total essential amino acids (TEAAs), total non-essential amino acids (TNEAAs), and total amino acids (TAAs) compared to SCK after the MeJA treatment. However, the MeJA (500 and 800 μM) treatments significantly increased the levels of TEAAs, TNEAAs, and TAAs in hydroponic Chinese chives compared to those in HCK. This effect was primarily due to a significant increase in the levels of EAAs (such as lysine, isoleucine, valine, and methionine) and NEAAS (such as glycine, aspartic acid, proline, and glutamic acid).

**TABLE 4:** Effect of MeJA treatments on the amino acid contents of substrate-grown and hydroponic Chinese chive.

Amino acid (mg g^–1^)	Substrate				Hydroponics			
	SCK	SM300	SM500	SM800	HCK	HM300	HM500	HM800
**Essential amino acids**								
Lysine	73.66 ± 1.92a	59.53 ± 1.01b	53.31 ± 1.01c	51.17 ± 1.27c	62.50 ± 0.93b	61.36 ± 1.14b	68.42 ± 0.88a	64.12 ± 2.52ab
Threonine	1.44 ± 0.02a	1.20 ± 0.03b	0.96 ± 0.01d	1.09 ± 0.05c	1.41 ± 0.01ab	1.35 ± 0.02b	1.46 ± 0.01a	1.46 ± 0.05a
Phenylalanine (ArAAs)	1.51 ± 0.02a	1.24 ± 0.02b	1.02 ± 0.02d	1.13 ± 0.02c	1.45 ± 0.01ab	1.4 ± 0.02b	1.51 ± 0.01a	1.50 ± 0.05a
Tryptophan (ArAAs)	0.34 ± 0.01a	0.35 ± 0.00a	0.28 ± 0.00c	0.31 ± 0.00b	0.47 ± 0.01a	0.35 ± 0.01b	0.32 ± 0.00c	0.29 ± 0.01d
Leucine	1.76 ± 0.03a	1.65 ± 0.02a	1.32 ± 0.14b	1.52 ± 0.02ab	1.65 ± 0.02a	1.33 ± 0.09b	1.76 ± 0.01a	1.66 ± 0.04a
Isoleucine	1.84 ± 0.04a	1.76 ± 0.02ab	1.47 ± 0.02c	1.67 ± 0.06d	1.75 ± 0.02b	1.50 ± 0.04c	1.86 ± 0.01a	1.73 ± 0.05b
Valine	2.28 ± 0.04a	1.92 ± 0.03b	1.57 ± 0.08c	1.73 ± 0.08bc	2.12 ± 0.03b	1.70 ± 0.03c	2.40 ± 0.01a	2.24 ± 0.10ab
Methionine (SAAs)	1.76 ± 0.02ab	1.90 ± 0.04a	1.70 ± 0.02b	1.52 ± 0.08c	1.14 ± 0.02c	1.11 ± 0.03c	1.85 ± 0.01a	1.72 ± 0.04b
**Non-essential amino acids**								
Cysteine (SAAs)	0.01 ± 0.00b	0.04 ± 0.00a	0.03 ± 0.00a	0.04 ± 0.01a	0.01 ± 0.00a	0.01 ± 0.00a	0.02 ± 0.00a	0.01 ± 0.00a
Alanine	8.07 ± 0.19b	6.73 ± 0.16c	4.87 ± 0.05d	9.26 ± 0.06a	9.54 ± 0.08bc	12.43 ± 0.12a	10.17 ± 0.02b	8.93 ± 0.48c
Glycine	1.10 ± 0.05a	0.82 ± 0.03b	0.70 ± 0.04b	0.81 ± 0.06b	0.92 ± 0.02c	1.08 ± 0.02bc	1.13 ± 0.02b	1.46 ± 0.11a
Tyrosine (ArAAs)	0.60 ± 0.00a	0.45 ± 0.02b	0.31 ± 0.01c	0.41 ± 0.02b	0.78 ± 0.00a	0.79 ± 0.03a	0.54 ± 0.01b	0.58 ± 0.03b
Asparagine	1.51 ± 0.05a	1.55 ± 0.07a	1.11 ± 0.09b	1.39 ± 0.04a	1.36 ± 0.02bc	1.24 ± 0.03c	1.50 ± 0.05a	1.48 ± 0.05ab
Proline	6.62 ± 0.09a	6.41 ± 0.09a	5.70 ± 0.10b	5.59 ± 0.22b	5.94 ± 0.08b	5.66 ± 0.06b	6.93 ± 0.10a	6.73 ± 0.19a
Serine	2.96 ± 0.07a	2.29 ± 0.05b	1.70 ± 0.04c	1.33 ± 0.09d	2.37 ± 0.04a	2.23 ± 0.06a	2.39 ± 0.03a	2.47 ± 0.14a
Glutamate	26.02 ± 0.71a	22.58 ± 0.49b	17.90 ± 0.26c	15.95 ± 0.78d	18.72 ± 0.36c	19.57 ± 0.26c	25.05 ± 0.86b	28.06 ± 1.21a
Glutamine	10.62 ± 0.30a	8.40 ± 0.14b	7.58 ± 0.17c	7.10 ± 0.09c	8.92 ± 0.25ab	8.76 ± 0.13b	9.68 ± 0.14a	9.07 ± 0.41ab
Arginine	12.09 ± 0.20a	10.08 ± 0.09b	9.59 ± 0.14b	8.77 ± 0.36c	12.48 ± 0.25a	13.71 ± 0.55a	12.58 ± 0.76a	11.83 ± 0.54a
Total essential amino acids (TEAAs)	84.57 ± 2.08a	69.56 ± 1.15b	61.62 ± 1.12c	60.13 ± 1.46c	72.48 ± 0.95b	70.07 ± 1.17b	79.59 ± 0.88a	74.73 ± 2.85ab
Total non-essential amino acids (TNEAAs)	69.59 ± 1.62a	59.37 ± 1.05b	49.50 ± 0.6c	50.66 ± 1.29c	61.02 ± 0.92b	65.48 ± 0.71ab	69.97 ± 0.63a	70.62 ± 3.13a
Total aromatic amino acids (TArAAs)	2.44 ± 0.04a	2.04 ± 0.04b	1.61 ± 0.03d	1.85 ± 0.03c	2.71 ± 0.01a	2.54 ± 0.02b	2.38 ± 0.01c	2.37 ± 0.08c
Total sulfur amino acids (TSAAs)	1.76 ± 0.03ab	1.93 ± 0.04a	1.74 ± 0.02b	1.56 ± 0.09c	1.15 ± 0.02c	1.12 ± 0.03c	1.87 ± 0.01a	1.73 ± 0.04b
(EAAs: TAAs) * 100 (%)	54.86 ± 0.06b	53.95 ± 0.04c	55.44 ± 0.19a	54.28 ± 0.04c	54.29 ± 0.31a	51.69 ± 0.52b	53.21 ± 0.27a	51.42 ± 0.16b
Total amino acids (TAAs)	154.16 ± 3.69a	128.94 ± 2.2b	111.12 ± 1.7c	110.79 ± 2.74c	133.50 ± 1.68c	135.55 ± 1.31bc	149.56 ± 1.29a	145.34 ± 5.97ab

*Data represent the mean ± standard error (n = 3). Different lowercase letters indicate statistical significance by Duncan’s multiple range test (p < 0.05).*

Most amino acids are aliphatic amino acids and function as precursors to aromatic compounds (54). Phenylalanine, tryptophan, and tyrosine are aromatic amino acids that participate in the shikimate pathway, which plays an important role in the aroma development in fruits (55, 56). We found that total aromatic amino acids were inhibited to varying degrees by the MeJA treatment in both substrate-grown and hydroponic Chinese chives. This was probably because MeJA treatment accelerates amino acid deamination and decarboxylation, and degradation of proteins and heat-sensitive amino acids (such as tyrosine) (57). Further studies are required to determine the physiological and molecular mechanisms underlying the effects of MeJA on amino acid synthesis and degradation. Sulfur-containing amino acids are known to play an important role in the production of flavor substances in the Maillard reaction (58). The characteristic flavor and aroma of Chinese chives is produced by the alliinase-catalyzed hydrolysis of metabolites, such as S-alk (alkene) cysteine sulfoxides during cell rupture (59). Volatile components mainly include organosulfur compounds, of which methionine produces methyl sulfide as the main bioactive substance. Methyl sulfide provides the pungent aroma of Chinese chives (60), rather than the fruity aroma provided by aromatic amino acids. We showed that the MeJA treatment significantly increased the cysteine content (sulfur amino acids) in the substrate-grown Chinese chives; moreover, treatment with 500 and 800 μM MeJA significantly increased the methionine content (51–62%) of hydroponic Chinese chives. This suggested that the sulfur-containing amino acids are the primary reason for the flavor of Chinese chives. In addition, sulfur-containing amino acids have an antioxidant capacity comparable to that of tert-butylhydroquinone under deep-frying conditions, and can therefore, be added to foods as antioxidants to extend their shelf life (61).

### Effect of Methyl Jasmonate Treatment on Primary Metabolite Profiles in Chinese Chive

**FIGURE 3:**
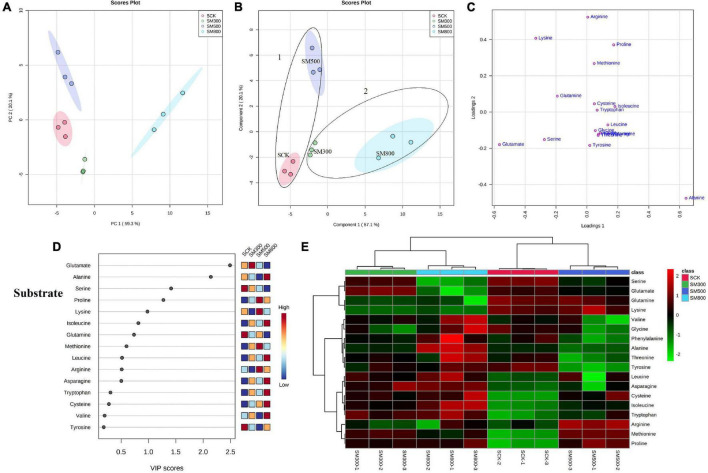
Statistical analyses of bioactive metabolites in substrate-grown Chinese chive treated with methyl jasmonate (MeJA). **(A)** An unsupervised principal component analysis (PCA) score plot of amino acid metabolites. **(B)** A partial least squares discriminant analysis (PLS-DA) score plot of amino acid metabolites. **(C)** PLS-DA score plots loaded with different amino acid metabolites. **(D)** VIP scores of amino acid metabolites in PLS-DA. The scores from low to high determine the importance of variables. The colored boxes on the right show the relative concentration of each metabolite from low (blue) to high (red). **(E)** Heat map of amino acid concentrations. The colored areas correspond to the concentrations of different amino acids from low (green) to high (red) in groups treated with different concentrations of MeJA. Each row represents an amino acid, and each column represents a treatment.

**FIGURE 4:**
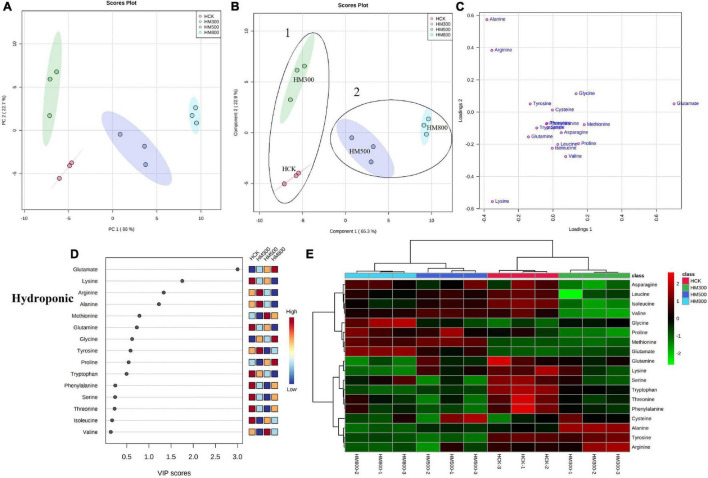
Statistical analyses of bioactive metabolites in hydroponic Chinese chive treated with methyl jasmonate (MeJA). **(A)** An unsupervised principal component analysis (PCA) score plot of amino acid metabolites. **(B)** A partial least squares discriminant analysis (PLS-DA) score plot of amino acid metabolites. **(C)** PLS-DA score plots loaded with different amino acid metabolites. **(D)** VIP scores of amino acid metabolites in PLS-DA. The scores from low to high determine the importance of variables. The colored boxes on the right show the relative concentration of each metabolite from low (blue) to high (red). **(E)** Heat map of amino acid concentrations. The colored areas correspond to the concentrations of different amino acids from low (green) to high (red) in groups treated with different concentrations of MeJA. Each row represents an amino acid, and each column represents a treatment.

An unsupervised principal component analysis (PCA) of primary metabolites (amino acids) using UPLC-MS/MS data confirmed a high degree of reproducibility between the three biological replicates and treatments. These results helped to determine that the MeJA-treated substrate-grown or hydroponic Chinese chives contained the most and least amino acid metabolites, respectively (Figures 3A, 4A). The PC1 and PC2 axes for the substrate-grown Chinese chives explained 79.4% of the total variance (59.3 and 20.1%, respectively) (Figure 3A), and those for hydroponically grown Chinese chives explained 88.7% of the total variance (66 and 22.7%, respectively) (Figure 4A). The PCA score plot clearly distinguished the MeJA-treated and control groups in a systematic manner according to the amino acids in Chinese chives. This indicated that treatment with different concentrations of MeJA had a considerable effect on the amino acid content of Chinese chives.

To obtain more specific and meaningful information regarding the metabolites, we used PLS-DA to determine the metabolite changes in Chinese chives treated with different concentrations of MeJA. PLS-DA uses a general algorithm based on reduced dimensional discriminant analysis, which provides predictive and descriptive analyses for the selection of discriminant variables (62). This analysis has the advantage of not relying on specific distributions, resulting in more accurate predictive and descriptive models (63). We found that the PLS-DA models for substrate-grown and hydroponic Chinese chives fitted well (*R*^2^ = 0.97 and 0.99, respectively) and were highly predictable (*Q*^2^ = 0.88 and 0.96, respectively), thus, allowing us to predict the changes in amino acid metabolites. The PC1 and PC2 axes of the substrate-grown Chinese chives explained 77.2% of the total variance (57.1 and 20.1%, respectively) (Figure 3B), and those of the hydroponic Chinese chives explained 88.2% of the total variance (65.3 and 22.9%, respectively) (Figure 4B). In the PLS-DA score plot, the substrate-grown or hydroponic Chinese chives treated with different concentrations of MeJA were divided into two major groups according to the levels of 18 amino acids. Thus, different concentrations of MeJA and control treatments had significant effects on the amino acid content of the substrate-grown or hydroponic Chinese chives. Based on the proximity of data points, the SCK and SM500 groups of the substrate-grown Chinese chives showed similar amino acid metabolisms. The PLS-DA loading plots indicated that the greater the distance between the metabolite points and the origin, the greater the contribution of the metabolite to the total variation (64) (Figures 3C, 4C). Among the substrate-grown Chinese chives, lysine and glutamine were positively loaded on PC2 and separated the 500 μM MeJA-treated group from other treatment groups. Glycine, valine, phenylalanine, threonine, leucine, tyrosine, and alanine were positively loaded on PC1 and separated the 800 μM MeJA-treated group from other treatment groups. Serine and glutamate showed negative loading on PC1 and PC2 and separated HCK and the 300 μM MeJA-treated group from other treatment groups (Figures 3B,C). Therefore, these 11 amino acid metabolites explained most of the variations between substrate-grown Chinese chives treated with different concentrations of MeJA. Among the hydroponic plants, glutamate and glycine were positively loaded on PC1, separating the 800 μM MeJA-treated group from the other treatment groups. Alanine, arginine, and tyrosine were positively loaded on PC2, separating the 300 μM MeJA-treated group from the other treatment groups. Lysine, glutamine, tryptophan, serine, phenylalanine, and threonine were loaded on PC1 and PC2, separating HCK from other treatment groups. Threonine, leucine, proline, asparagine, and methionine were positively loaded on PC1 and PC2, separating the 800 μM MeJA-treated group from the other treatment groups (Figures 4B,C). Therefore, these 16 amino acid metabolites explained most of the variations between the hydroponic Chinese chives treated with different concentrations of MeJA.

To further elucidate the amino acid metabolites in Chinese chives treated with different concentrations of MeJA, we performed a hierarchical cluster analysis using the entire dataset of identified metabolites. The results yielded different clusters of samples with similar chemical composition, and we prepared a heat map based on metabolite concentrations in all samples. The heat map includes the data of the 18 amino acid metabolites identified in the substrate-grown or hydroponic Chinese chives (Figures 3E, 4E). To some extent, the hierarchical cluster analysis confirmed the two main clusters observed in the PLS-DA score plots (Figures 3B, 4B), which can be inferred from the branches at the top of the heat map (Figures 3E, 4E). In addition, the heat map indicated the trends of amino acid metabolic composition in the substrate-grown or hydroponic Chinese chives treated with different concentrations of MeJA. The substrate-grown Chinese chives in the SCK group contained higher levels of serine and glutamine, the 300 μM MeJA-treated group contained higher levels of glutamate, the 500 μM MeJA-treated group contained higher levels of arginine; and the 800 μM MeJA-treated group contained higher levels of alanine (Figure 3E). The HCK treatment group of hydroponic Chinese chives contained high levels of threonine, phenylalanine, and tryptophan, the 300 μM MeJA-treated group contained high levels of alanine, the 500 μM MeJA-treated group contained high levels of cysteine and methionine, and the 800 μM MeJA-treated group contained high levels of glycine and glutamate (Figure 4E).

In the PLS-DA analysis, we used the VIP scores to measure the strength of influence and explanatory power of each amino acid metabolite on the categorical discrimination of the sample. Higher VIP scores indicate greater differences between treatments, and are useful for selecting biomarkers that differ between treatments (48). To provide the most meaningful interpretation of the results, we considered only the top metabolites with VIP scores > 1 (65) (Figures 3D, 4D). The top four amino acid metabolites (VIP scores > 1) in the substrate-grown Chinese chives were (from highest to lowest) glutamate, alanine, serine, and proline, which had the highest relative concentrations in the SM300, SM800, SCK, and SM500 treatments, respectively. These patterns allowed us to distinguish between the treatments (Figure 3D). The top four amino acid metabolites (VIP scores > 1) in the hydroponic Chinese chives were glutamate, lysine, arginine, and alanine. Glutamate and lysine showed the highest relative concentrations in the HM800 and HCK treatments, respectively, whereas arginine and alanine showed the highest relative concentration in the HM300 treatment (Figure 4D). The higher VIP scores for these compounds suggested that they are important biomarkers for describing variations in the primary metabolites in Chinese chives.

### Effect of Methyl Jasmonate Treatments on Phenolic Compounds (Secondary Metabolites) and Total Phenol Content in Chinese Chive

The levels of 15 phenolic compounds in the substrate-grown and hydroponic Chinese chives treated with MeJA are listed in Table 5. The MeJA treatment significantly increased (2.8–47%) the phenolic content of the substrate-grown Chinese chives compared with that of SCK, and the 500 μM MeJA treatment had the most significant effect. This was consistent with our previous study, where the application of 500 μM MeJA increased the total phenol contents in Chinese chives (66). The phenolic compounds of hydroponic Chinese chives treated with 500 and 800 μM MeJA were significantly higher than those of the HCK group. Phenolic compounds are known to benefit human health, and can be used as functional foods (67). Phenolics includes coumarins, phenolic acids, such as hydroxybenzoic acids (68) and hydroxycinnamic acids (69), flavonoids, such as flavonols (70), flavones (71), flavanols (72), flavanones (73), isoflavones, anthocyanins, chalcones and non-flavonoids, such as tannins, lignans, and stilbenes. Flavonoids, which are widely found in plants, have antioxidant, anticancer, antibacterial, and antimutagenic activities (74), and exhibit high levels of antioxidant activity as plant secondary metabolites (75). Among the substrate-grown Chinese chives, the total phenolic acid content was significantly lower in the MeJA-treated groups; however, the content of total flavonoids in the MeJA-treated groups was significantly higher than that in the control group. In the hydroponic plants, the total flavonoid and total phenolic acid contents of groups treated with 300 and 500 μM MeJA were significantly higher than those of the control group. This suggested that the effect of the MeJA treatment on the flavonoid and phenolic acid contents of Chinese chives depended mainly on the cultivation methods. Baek et al. reported similar results after applying MeJA treatment to pak choi (*Brassica rapa* L. ssp. *chinensis*) in soil and hydroponic systems (76). Chinese chives contain high levels of ferulic acid, which has a strong antioxidant capacity and can lower triglyceride levels (77). Protocatechuic acid and rutin have strong free radical scavenging abilities and inhibit lipid peroxidation (78, 79). In the present study, the MeJA treatment in substrate-grown Chinese chives significantly increased the levels of phenolic acids, such protocatechuic acid (6.0–33%), p-hydroxybenzoic acid (1.4–12%), chlorogenic acid (3.8–4.5%), ferulic acid (0.4–4.1%), and erucic acid (5.1–12.5%), and flavonoids, such as rutin (23–351%). In addition, the levels of cinnamic acid, gentilic acid, caffeic acid, cynarin, kaempferol, and quercetin also increased to different degrees. Among the hydroponic Chinese chives, treatment with MeJA (500 and 800 μM) significantly increased the levels of protocatechuic acid (20 and 404%, respectively), chlorogenic acid (2.0 and 4.1%, respectively), and 4-coumaric acid (12 and 22%, respectively) compared with those in the HCK treatment. Treatment with a low concentration of MeJA (300 μM) also significantly increased the levels of 4-coumaric acid, gentilic acid, caffeic acid, cynarin, rutin, and quercetin. These results indicated that the MeJA-treated substrate-grown Chinese chives had higher levels of most phenolic components compared to the hydroponic Chinese chives. In addition, the changes in the total phenol content of MeJA-treated substrate-grown and hydroponic Chinese chives showed similar trends to those of the total phenolic compounds detected by HPLC (Supplementary Figure 6).

**TABLE 5:** Effect of MeJA treatments on the phenolic component contents of substrate-grown and hydroponic Chinese chive.

Phenolic components (μ g g^–1^)	Substrate				Hydroponics			
	SCK	SM300	SM500	SM800	HCK	HM300	HM500	HM800
**Phenolic acids**								
Protocatechuic acid	90.39 ± 0.21d	102.07 ± 0.22b	120.44 ± 1.16a	95.78 ± 0.53c	91.18 ± 0.39c	94.44 ± 1.03c	109.03 ± 1.51b	459.32 ± 7.33a
P-hydroxybenzoic acid	79.83 ± 0.04c	89.45 ± 0.21a	81.25 ± 0.05b	80.93 ± 0.04b	81.20 ± 0.07a	81.02 ± 0.07a	79.76 ± 0.1b	79.85 ± 0.02b
Chlorogenic acid	97.03 ± 0.18c	100.72 ± 0.07b	101.38 ± 0.07a	100.85 ± 0.05b	96.08 ± 0.04c	95.95 ± 0.14c	97.97 ± 0.08b	100.05 ± 0.48a
Gallic acid	288.80 ± 0.17a	216.78 ± 1.33c	216.11 ± 0.86c	247.42 ± 1.13b	270.66 ± 11.08ab	216.30 ± 8.08c	264.88 ± 3.07b	293.54 ± 2.67a
4-Coumaric acid	83.71 ± 0.06a	82.39 ± 0.09b	83.85 ± 0.04a	82.14 ± 0.06c	83.73 ± 0.07c	103.64 ± 0.99a	94.18 ± 0.64b	101.86 ± 0.76a
Ferulic acid	86.71 ± 0.04d	87.43 ± 0.13b	87.07 ± 0.08c	90.30 ± 0.08a	207.99 ± 1.97a	191.33 ± 1.76c	174.73 ± 4.64b	183.08 ± 3.08bc
Benzoic acid	133.04 ± 0.71a	91.05 ± 0.08d	101.90 ± 0.13c	113.11 ± 0.52b	237.68 ± 2.73a	105.34 ± 4.97b	102.07 ± 0.65b	106.26 ± 0.77b
Cinnamic acid	93.61 ± 0.04c	95.00 ± 0.17b	93.79 ± 0.04c	96.33 ± 0.35a	95.09 ± 0.18a	94.23 ± 0.06b	93.54 ± 0.13c	94.71 ± 0.17a
Gentilic acid	103.00 ± 0.12b	99.68 ± 0.03d	108.68 ± 0.30a	101.68 ± 0.07c	107.55 ± 0.61c	111.42 ± 0.32a	109.70 ± 0.76b	101.43 ± 0.14d
Caffeic acid	89.07 ± 0.03b	89.88 ± 0.00a	87.77 ± 0.03d	88.35 ± 0.06c	88.43 ± 0.12b	93.00 ± 0.17a	88.80 ± 0.15b	93.12 ± 0.12a
Cynarin	88.28 ± 0.06b	88.86 ± 0.02a	88.32 ± 0.05b	88.75 ± 0.04a	96.68 ± 0.22c	107.00 ± 0.31a	95.77 ± 0.33c	103.49 ± 0.53b
Erucic acid	100.80 ± 0.75d	108.99 ± 0.65b	113.45 ± 0.43a	105.99 ± 0.35c	134.62 ± 2.10ab	133.10 ± 2.09b	114.08 ± 1.01c	140.00 ± 2.20a
**Flavones**								
Kaempferol	173.32 ± 0.44b	168.21 ± 0.15d	169.11 ± 0.13c	178.92 ± 0.19a	173.65 ± 0.96b	174.38 ± 1.20b	175.17 ± 0.66b	188.12 ± 3.13a
Rutin	255.78 ± 3.94d	614.30 ± 1.14b	1153.23 ± 3.02a	313.61 ± 1.33c	312.12 ± 2.71b	382.69 ± 10.33a	227.29 ± 3.31c	318.18 ± 2.69b
Quercetin	352.63 ± 0.81c	348.82 ± 0.54d	511.88 ± 0.94a	390.54 ± 0.57b	388.00 ± 2.33b	666.91 ± 37.86a	332.10 ± 2.12b	353.11 ± 7.23b
Total phenolic acids	1334.26 ± 1.96a	1252.29 ± 1.11d	1284.03 ± 0.22c	1291.62 ± 2.41b	1590.89 ± 7.90b	1426.78 ± 16.90c	1424.48 ± 4.5c	1856.71 ± 14.28a
Total flavones	781.73 ± 3.27d	1131.33 ± 0.47b	1834.22 ± 2.83a	883.07 ± 2.08c	873.76 ± 3.89b	1223.98 ± 28.93a	734.56 ± 3.66c	859.41 ± 12.72b
Total	2115.99 ± 1.45d	2383.62 ± 0.98b	3118.25 ± 2.96a	2174.69 ± 3.61c	2464.65 ± 11.46b	2650.76 ± 35.11a	2159.04 ± 2.07c	2716.12 ± 25.37a

*Data represent the mean ± standard error (n = 3). Different lowercase letters indicate statistical significance by Duncan’s multiple range test (p < 0.05).*

### Effect of the Methyl Jasmonate Treatment on Secondary Metabolite Profiles in Chinese Chive

**FIGURE 5:**
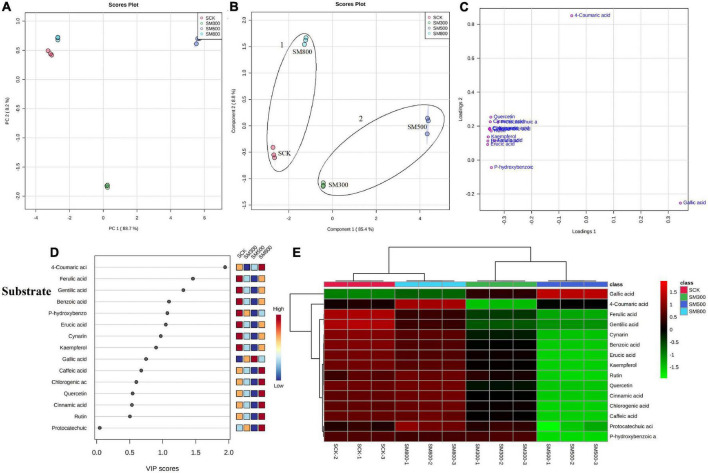
Statistical analyses of bioactive metabolites in substrate-grown Chinese chive treated with methyl jasmonate (MeJA). **(A)** An unsupervised principal component analysis (PCA) score plot of phenolic metabolites. **(B)** A partial least squares discriminant analysis (PLS-DA) score plot of phenolic metabolites. **(C)** PLS-DA score plots loaded with different phenolic metabolites. **(D)** VIP scores of phenolic metabolites in PLS-DA. The scores from low to high determine the importance of variables. The colored boxes on the right show the relative concentration of each metabolite from low (blue) to high (red). **(E)** Heat map of phenolic concentrations. The colored areas correspond to the concentrations of different amino acids from low (green) to high (red) in groups treated with different concentrations of MeJA. Each row represents a phenolic, and each column represents a treatment.

**FIGURE 6:**
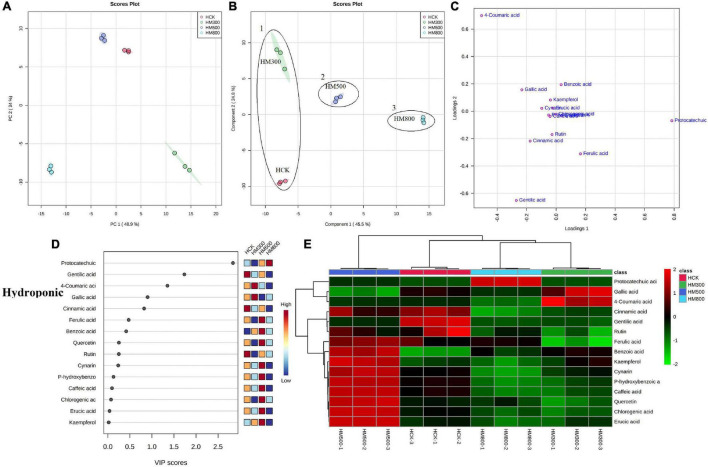
Statistical analyses of bioactive metabolites in hydroponic Chinese chive treated with methyl jasmonate (MeJA). **(A)** An unsupervised principal component analysis (PCA) score plot of phenolic metabolites. **(B)** A partial least squares discriminant analysis (PLS-DA) score plot of phenolic metabolites. **(C)** PLS-DA score plots loaded with different phenolic metabolites. **(D)** VIP scores of phenolic metabolites in PLS-DA. The scores from low to high determine the importance of variables. The colored boxes on the right show the relative concentration of each metabolite from low (blue) to high (red). **(E)** Heat map of phenolic concentrations. The colored areas correspond to the concentrations of different amino acids from low (green) to high (red) in groups treated with different concentrations of MeJA. Each row represents a phenolic, and each column represents a treatment.

The PCA analysis showed that the PC1 and PC2 axes for the substrate-grown Chinese chives explained 96.9% of the total variance (88.7 and 8.2%, respectively) (Figure 5A), and those for hydroponic plants explained 82.9% of the total variance (48.9 and 34%, respectively) (Figure 6A). The PCA score plot distinguished the MeJA-treated groups from controls systematically and clearly based on the phenolic compounds in Chinese chives. The PLS-DA score plot showed that the PC1 and PC2 axes for the substrate-grown Chinese chives explained 94.2% of the total variance (85.4 and 8.8%, respectively) (Figure 5B), and those for hydroponic Chinese chives explained 70.3% of the total variance (45.5 and 24.8%, respectively) (Figure 6B). Based on the sample distribution in the PLS-DA plot, we hypothesized that there are two large groups in the substrate-grown Chinese chives and three large groups in the hydroponic Chinese chives (Figures 5B, 6B). Based on the proximity of data points, the SCK and SM800 groups of the substrate-grown Chinese chives showed similar phenolic metabolism and were different from the SM300 and SM500 groups. Among the hydroponic Chinese chives, the HCK and HM300 groups had similar phenolic metabolism. Further, among the substrate-grown Chinese chives, the metabolite 4-coumaric acid was loaded positively on PC2, gallic acid was loaded positively on PC1, and p-hydroxybenzoic acid was loaded negatively on PC1 and PC2. Thus, these three phenolic metabolites separated the treatment groups of the substrate-grown Chinese chives (Figures 5B,C) and explained most of the variation in plants treated with different MeJA concentrations. For the hydroponic Chinese chives, 4-coumaric acid, gallic acid, kaempferol, cynarin, and erucic acid were negatively loaded on PC1; gentilic acid, cinnamic acid, rutin, caffeic acid, and p-hydroxybenzoic acid were negatively loaded on PC2; and benzoic acid, protocatechuic acid, and ferulic acid were positively loaded on PC1. Thus, these 13 phenolic metabolites separated the various treatment groups of the hydroponic Chinese chives (Figures 6B,C) and explained most of the variation between plants treated with different concentrations of MeJA. The hierarchical clustering heat map showed 15 phenolic compound metabolites identified in the substrate-grown or hydroponic cultures of Chinese chives (Figures 5E, 6E). The SCK group contained higher levels of amino acids than other substrate-grown Chinese chives (Figure 5E), and the HM500 treatment group of hydroponic plants contained a higher level of amino acids than other hydroponics (Figure 6E). The VIP scores plot showed that in the substrate-grown Chinese chives, the top six phenolic metabolites (VIP scores > 1) were 4-coumaric acid, ferulic acid, gentilic acid, benzoic acid, p-hydroxybenzoic acid, and erucic acid (Figure 5D). In the hydroponic Chinese chives, the top three phenolic metabolites (VIP scores > 1) were protocatechuic acid, gentilic acid, and 4-coumaric acid (Figure 6D). The higher VIP scores for these compounds suggested that they were important biomarkers for describing variations in the phenolic compounds of the primary metabolites in Chinese chives.

### Effect of the Methyl Jasmonate Treatments on Carotenoids (Secondary Metabolites) in Chinese Chive

Carotenoid is a lipophilic antioxidant and can detoxify various forms of reactive oxygen species (ROS) (80). The levels of different carotenoids in the substrate-grown and hydroponic Chinese chives treated with MeJA are listed in Table 6. β-carotene showed the highest levels (2027.16 and 1466.89 μg g^–1^) in the substrate-grown and hydroponic Chinese chives, followed by lycopene (223.74 and 121.46 μg g^–1^, respectively), lutein (159.21 and 196.54 μg g^–1^, respectively) and monocyclic zeaxanthin (77.43 and 63.58 μg g^–1^, respectively). Carotenoids include, β-carotene, α-carotene (81), and xanthophylls, such as zeaxanthin, neoxanthin, violaxanthin, lutein (82), and lycopene, which have antioxidants capacity (83, 84). The carotenoids in Chinese chives mainly included β-carotene. Lutein enhances the immune function and improves the antioxidant capacity of the body, protects vision, and reduces the severity of age-related macular degeneration (64, 85). The levels of most carotenoids, such as lutein, violaxanthin, zeaxanthin, and α-carotene, were significantly higher in the substrate-grown Chinese chives treated with MeJA (500 μM) than in the SCK group. Treatment with 800 μM MeJA significantly increased the total carotenoid content (37%), mainly because this concentration significantly increased the most abundant β-carotene in Chinese chives (48%). These results were similar to those of Pérez et al. (86). Previous studies have shown that β-carotene content is genotypically determined (64). However, we found that the β-carotene content in the substrate-grown Chinese chives was significantly higher than that in the hydroponic Chinese chives with or without MeJA treatment, suggesting that cultivation method is also a key factor in determining the β-carotene content in Chinese chives. A study on tomato fruits reported that JA treatment had a dose-dependent effect on carotenoid accumulation; that is, when the concentration of JA was > 0.5 μM, carotenoid biosynthesis was inhibited in tomato (31). In our study, hydroponic Chinese chives treated with MeJA (300 μM) showed significantly higher levels of zeaxanthin, α-carotene, and lutein compared to those in the HCK group. Treatment with 500 μM MeJA significantly increased the levels of violet xanthin. However, the carotenoid content in the hydroponic Chinese chives treated with MeJA (500 μM) showed an overall decreasing trend. In addition, the carotenoid content in the substrate-grown Chinese chives was higher than that in the hydroponic Chinese chives with or without MeJA treatment. This may have been because in leafy vegetables, carotenoid levels depend on various factors, such as cultivar species, cultivation method, maturity, and environmental conditions (87). Future studies should investigate the effect of different concentrations of MeJA on carotenoid biosynthesis to improve our understanding on the accumulation of carotenoids in hydroponic Chinese chives.

**TABLE 6:** Effect of MeJA treatments on the carotenoid contents of substrate-grown and hydroponic Chinese chive.

Carotenoids (μ g g^–1^)	Substrate				Hydroponics			
	SCK	SM300	SM500	SM800	HCK	HM300	HM500	HM800
Violaxanthin	29.71 ± 0.03b	30.61 ± 0.02a	30.67 ± 0.06a	30.65 ± 0.04a	30.24 ± 0.16bc	30.68 ± 0.27ab	31.12 ± 0.06a	29.57 ± 0.33c
Monoepoxy zeaxanthin	77.43 ± 0.48a	68.89 ± 1.52b	65.20 ± 0.18c	78.98 ± 0.6a	63.58 ± 0.55b	66.01 ± 0.66b	57.18 ± 0.25c	70.36 ± 1.65a
Lycopene	223.74 ± 0.29a	90.74 ± 2.94d	204.99 ± 5.03b	166.48 ± 0.94c	121.46 ± 2.19a	117.13 ± 4.84a	59.56 ± 2.98c	94.77 ± 0.93b
β-carotene	2027.16 ± 45.45b	1633.89 ± 37.22c	1648.63 ± 9.59c	2992.70 ± 52.49a	1466.89 ± 8.41a	1369.18 ± 30.71b	1126.55 ± 23.06c	1532.63 ± 32.15a
Zeaxanthin	16.79 ± 0.05c	25.85 ± 0.36b	46.39 ± 4.26a	17.63 ± 0.22c	7.45 ± 0.47c	14.62 ± 0.26a	4.60 ± 0.18d	9.56 ± 0.49b
α-carotene	6.03 ± 0.61d	25.41 ± 1.76c	65.59 ± 3.01a	34.72 ± 0.45b	20.06 ± 0.09b	29.23 ± 1.69a	17.34 ± 0.84b	17.01 ± 0.33b
Lutein	159.21 ± 3.85b	108.88 ± 3.60c	173.89 ± 3.69a	149.50 ± 2.85b	96.54 ± 1.40b	134.22 ± 7.04a	77.29 ± 0.26c	94.74 ± 1.4b
Total	2540.07 ± 48.92b	1984.27 ± 37.26d	2235.35 ± 11.62c	3470.66 ± 55.29a	1806.23 ± 12.07a	1761.07 ± 37.68a	1373.64 ± 24.79b	1848.64 ± 31.87a

*Data represent the mean ± standard error (n = 3). Different lowercase letters indicate statistical significance by Duncan’s multiple range test (p < 0.05).*

### Effect of the Methyl Jasmonate Treatments on the Antioxidant Capacities of Chinese Chive

**FIGURE 7:**
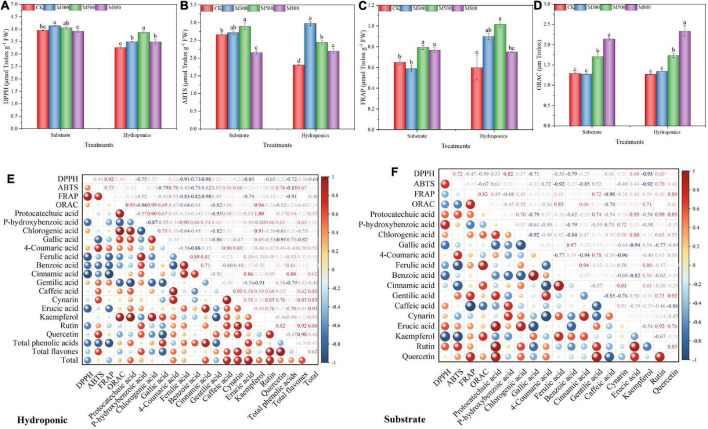
The 2,2-diphenyl-1-picrylhydrazyl (DPPH) radical scavenging capacity **(A)**, Trolox-equivalent antioxidant capacity (ABTS) **(B)**, ferric-reducing antioxidant power (FRAP) **(C)**, and oxygen radical absorbance capacity (ORAC) **(D)** of Chinese chives grown in substrate or hydroponics as affected by methyl jasmonate (MeJA) treatments. Data represent the mean ± standard error (*n* = 3). Different lowercase letters indicate statistical significance, by Duncan’s multiple range tests (*p* < 0.05). The heat map shows the Pearson correlation analysis between the observed parameters of antioxidant capacity and the observed parameters of phenolic compounds in MeJA treated hydroponic **(E)** or substrate **(F)** Chinese chive. The values in the heat map are Pearson’s correlation coefficient. The size and color depth of the colored balls correspond to the strength of the correlation, ranging from weak (small balls, blue) to strong (large balls, red).

The antioxidant activities in the substrate-grown and hydroponic Chinese chives treated with MeJA are listed in Figures 7A–D. The ABTS (Figure 7B), FRAP (Figure 7C), and ORAC (Figure 7D) assays showed that the antioxidant activities of the substrate-grown and hydroponic Chinese chives treated with MeJA (500 μM) were significantly higher than those in the SCK and HCK control groups, respectively. Exogenous MeJA can increase the antioxidant activity of plants (88), which is consistent with our findings. The DPPH activities of hydroponic Chinese chives treated with MeJA and of substrate-grown Chinese chives treated with 300 μM MeJA were significantly higher than that of the HCK group (Figure 7A). Both phenolics and flavonoids are strongly associated with antioxidant activity (DPPH and ABTS) in *Amaranthus hypochondriacus* (89), *A. tricolor* (84), *A. blitum* (90), weedy species (91), stem amaranth (92), green morph amaranth (93), and red morph amaranth (94), which are corroborative to the present findings. Phenolic compounds are usually positively associated with the antioxidant activity of plant foods (95). In addition, polyphenols have an ideal chemical structure for scavenging free radicals, and higher levels of polyphenols in plants are associated with stronger free radical scavenging ability (96, 97). The correlation heat map further confirmed the relationship between antioxidant activity and phenolic content of the substrate-grown (Figure 7F) and hydroponic (Figure 7E) Chinese chives. The correlation coefficients indicated the strength of the correlation between the two, and we considered only strong correlations by setting the threshold value to 0.5. In the substrate-grown Chinese chives, we observed strong positive correlations between DPPH activity and the levels of rutin, eruic acid, and p-hydroxybenzoic acid; ABTS and the levels of rutin, protocatechuic acid, and 4-coumaric acid; FRAP and the levels of quercetin and gentilic acid; and ORAC and the levels of kaempferol, cinnamic acid, chlorogenic acid, and ferulic acid (Figure 7F). In the hydroponic Chinese chives, we found positive correlations between ABTS activity and the levels of quercetin, cynarin, caffeic acid, gentilic acid, and 4-coumaric acid; FRAP and the level of 4-coumaric acid; and ORAC and the levels of kaempferol, gallic acid, and chloro-genic acid (Figure 7E). This further indicated that an increase in the level of phenolic compounds might be associated with the improved antioxidant capacity of the substrate-grown and hydroponic Chinese chives following the MeJA treatment. Following the MeJA treatment, the ORAC of the substrate-grown and hydroponic Chinese chives increased in a dose-dependent manner. This was similar to the trend of changes observed in the levels of phenolic compounds after the MeJA treatment. In addition to phenolic compounds, sulfur-containing compounds (66), vitamin C (98), chlorophylls (99), carotenoids (100), and polysaccharides (101) also showed strong antioxidant activity.

## Conclusion

Our results showed that the MeJA-treated Chinese chives showed significant differences in primary and secondary metabolites depending on the cultivation method. After treating the Chinese chives with MeJA, we distinguished between substrate-grown and hydroponic Chinese chives using amino acids and phenolic compounds. The MeJA treatment significantly increased the phenolic content of the substrate-grown Chinese chives and decreased the organic acids content of the hydroponic Chinese chives. Treatment with MeJA (500 μM) significantly increased the total sugar and amino acid (essential and non-essential amino acids and sulfur-containing amino acids) contents of hydroponically grown Chinese chives. However, the total sugar and amino acid contents of the substrate-grown Chinese chives showed trends contrasting to those of hydroponic Chinese chives. In addition, treatment with 500 μM MeJA significantly increased the antioxidant activity of both substrate-grown and hydroponic Chinese chives. The nutritional quality and bioactive substances of Chinese chives were comprehensively analyzed using metabolomics. The results of metabolites under different cultivation methods and MeJA treatments can contribute to increasing the nutritional quality and medicinal value of substrate-grown and hydroponic Chinese chives, and to some extent in promoting the flavor quality of Chinese chives, and promoting Chinese chives as a value-added horticultural product.

## Data Availability Statement

The original contributions presented in the study are included in the article/Supplementary Material, further inquiries can be directed to the corresponding author/s.

## Author Contributions

CW, JZ, JX, and JY conceived and designed the experiments. CW, JZ, and YG analyzed the data. CW wrote the manuscript. CW, JZ, JLi, TN, and JLv involved in the related discussion. JX, JZ, and BP improved the quality of the manuscript. All authors have read and agreed to the published version of the manuscript.

## Conflict of Interest

The authors declare that the research was conducted in the absence of any commercial or financial relationships that could be construed as a potential conflict of interest.

## Publisher’s Note

All claims expressed in this article are solely those of the authors and do not necessarily represent those of their affiliated organizations, or those of the publisher, the editors and the reviewers. Any product that may be evaluated in this article, or claim that may be made by its manufacturer, is not guaranteed or endorsed by the publisher.

## Footnotes

1
https://www.metaboanalyst.ca
