# Supplementary material for: Effect of Methyl Jasmonate Treatment on Primary and Secondary Metabolites and Antioxidant Capacity of the Substrate and Hydroponically Grown Chinese Chives
Source: Front Nutr. 2022 Apr 5;9:859035. doi: 10.3389/fnut.2022.859035 (PMC9016137; doi:10.3389/fnut.2022.859035)
Supplement: Supplementary file 1 [file Data_Sheet_1.pdf]

Supplementary Material

Supplementary Table S1

Supplementary Table S1. The limit of detection (LOD) and limit of quantification (LOQ) for the amino acid standards used in this study

Amino acid	Limit of detection (LOD) (ng mL^{-1})	Limit of quantification (LOQ) (ng mL^{-1})
Threonine	486.00	1620.00
Phenylalanine	843.60	2812.00
Leucine	733.20	2444.00
Isoleucine	1138.20	3794.00
Asparagine	119.10	397.00
Tryptophan	2246.10	7487.00
Methionine	6946.80	23156.00
Valine	1047.60	3492.00
Proline	2583.90	8613.00
Tyrosine	484.20	1614.00
Cysteine	365.40	1218.00
Alanine	283.80	946.00
Glycine	32.40	108.00
Serine	10.20	34.00
Glutamate	161.70	539.00
Arginine	553.80	1846.00
Glutamine	92.40	308.00
Lysine	111.60	372.00

Supplementary Table S2

Supplementary Table S2. Retention time, maximum absorption wavelength in visible region (λmax) and tentative identification of phenolic composition.

No.	Retention time (min)	Wavelengths of maximum absorption in the visible region (λmax)	Area (microvolts * s)	Height (microvolts)	Tentative identification
1	7.503	270 nm	1164582	60621	Gallic acid
2	13.302	259 nm	733475	67027	Protocatechuic acid
3	20.266	254 nm	672295	33807	P-hydroxybenzoic acid
4	22.07	238 nm	67271	4029	Chlorogenic acid
5	24.384	324 nm	725388	35669	Gentianic acid
6	26.657	322 nm	1375120	74632	Caffeic acid
7	30.83	240/322 nm	959925	50217	Cynarin
8	34.932	309 nm	2449463	145024	4-Coumaric acid
9	37.33	322 nm	1605539	103767	Ferulic acid
10	37.879	323 nm	646205	46204	Erucic acid
11	40.371	273 nm	172960	10645	Benzoic acid
12	45.688	255 nm	337199	27821	Rutin
13	50.409	276 nm	4164553	519669	Cinnamic acid
14	51.449	254 nm	2671052	345205	Quercetin
15	53.308	362 nm	917410	139027	Kaempferol

Supplementary Table S3

Supplementary Table S3. The limit of detection (LOD) and limit of quantification (LOQ) for the phenolic component standards used in this study

phenolic component	Limit of detection (LOD) (ng mL^{-1})	Limit of quantification (LOQ) (ng mL^{-1})
Protocatechuic acid		
P-hydroxybenzoic acid		
Chlorogenic acid		
Gentianic acid		
Caffeic acid		
Cynarin		
4-Coumaric acid	31.25	104.17
Ferulic acid		
Erucic acid		
Benzoic acid		
Rutin		
Cinnamic acid		
Quercetin		
Kaempferol	15.63	52.08

Supplementary Table S4

Supplementary Table S4. The limit of detection (LOD) and limit of quantification (LOQ) for the Carotenoids standards used in this study

Carotenoids	Limit of detection (LOD) (ng mL^{-1})	Limit of quantification (LOQ) (ng mL^{-1})
β-carotene	15.00	50.00
Zeaxanthin	3.00	10.00
α-carotene	3.00	10.00
Lutein	15.00	50.00
Violaxanthin	1.20	4.00
Monoepoxy zeaxanthin	1.20	4.00
Lycopene	15.00	50.00

Supplementary Figure S1

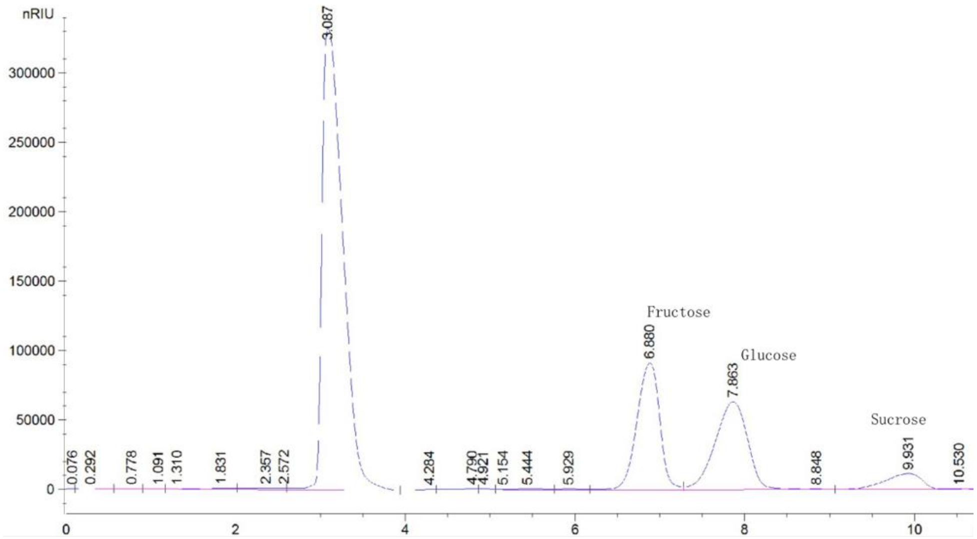

Supplementary Figure S1. Sugar chromatogram.

Supplementary Figure S2

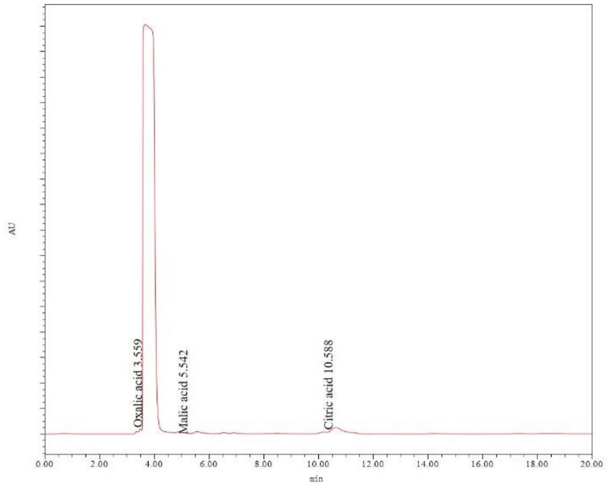

Supplementary Figure S2. Organic acid chromatogram.

Supplementary Figure S3

A

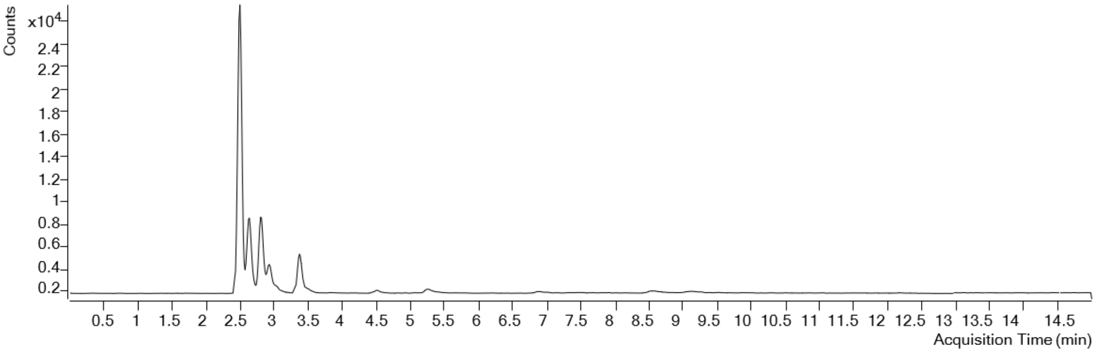

B

Compound	Threonine	Phenylalanin	Leucine	Isoleucine	Asparagine	Tryptophan	Methionine	Valine	Proline
Retention time (min)	2.493	2.499	2.644	2.808	2.82	2.928	3.033	3.381	3.394
Compound	Tyrosine	Cysteine	Alanine	Glycine	Serine	Glutamate	Arginine	Glutamine	Lysine
Retention time (min)	3.499	3.925	4.523	5.069	5.283	6.873	8.569	9.117	9.7

Supplementary Figure S3. Amino acid chromatogram (**A**) and amino acid retention time (**B**).

Supplementary Figure S4

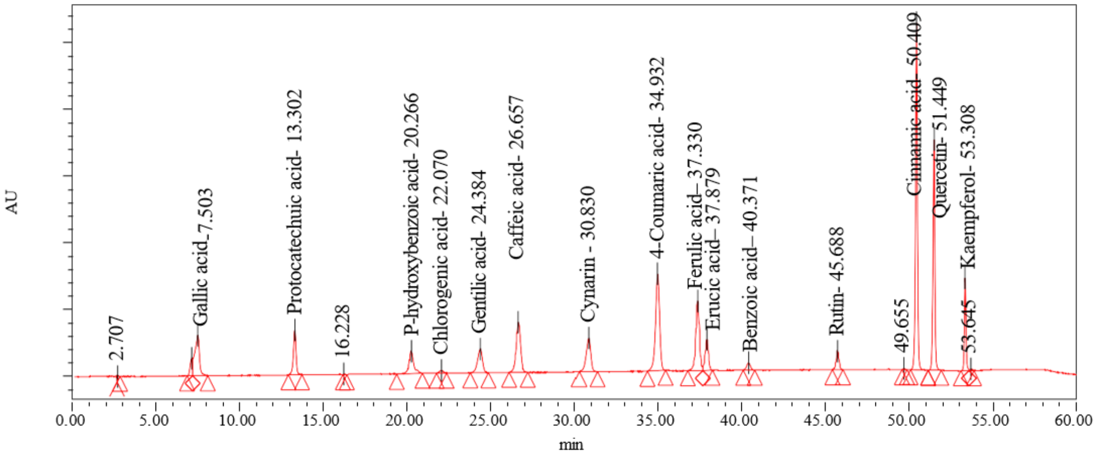

Supplementary Figure S4. Phenolic composition chromatogram.

Supplementary Figure S5

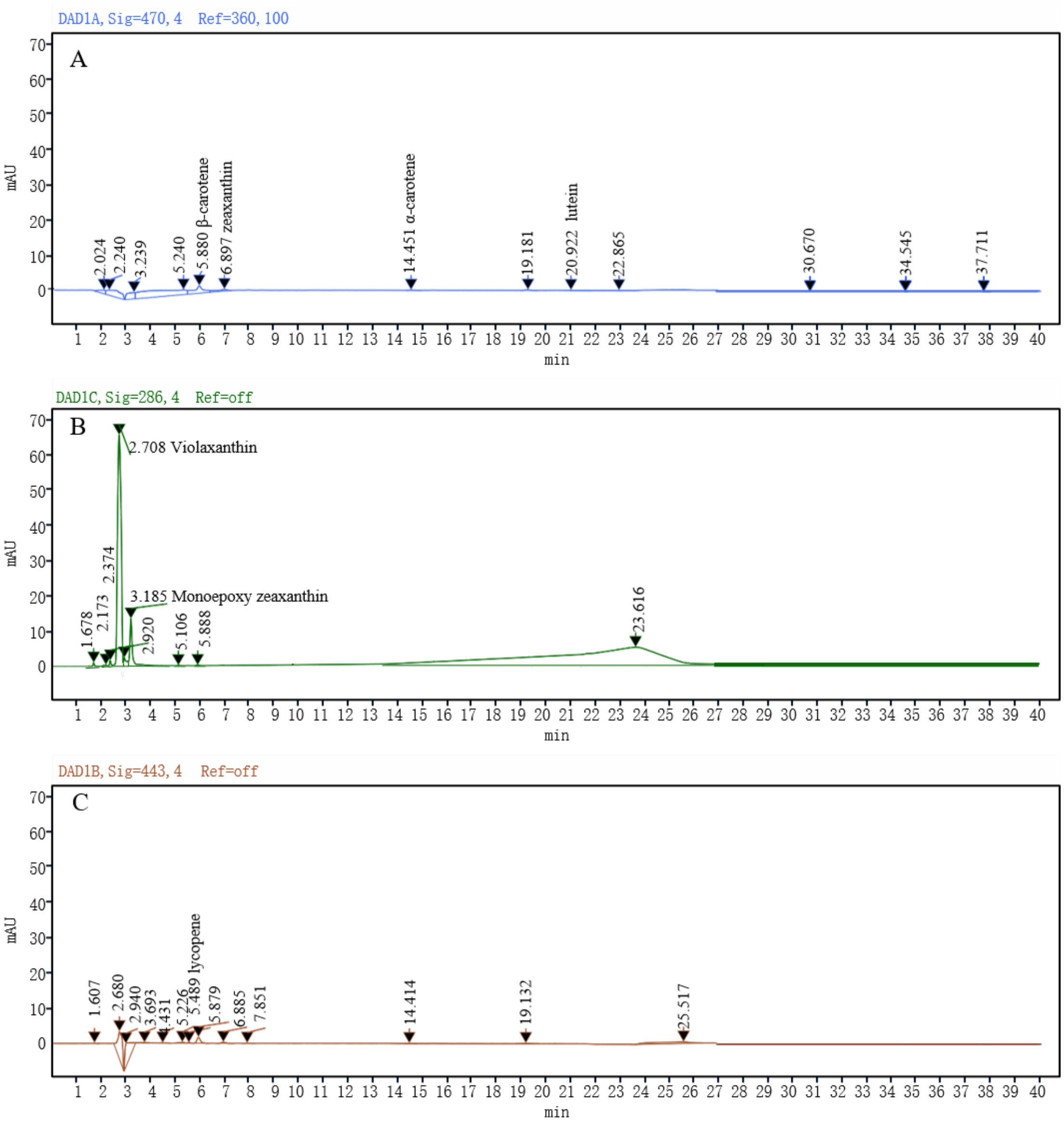

Supplementary Figure S5. Chromatograms of carotenoids detected at 470nm (**A**), 286nm (**B**) and 443nm (**C**)

Supplementary Table S5

Supplementary Table S5. Effect of MeJA treatments on marketable yield of substrate-grown Chinese chive.

Trentments	Fresh Weight (g $plant^{-1}$)	Marketable Yield (kg/m^2)	Marketable Yield (kg ha^{-1})	Increase in Yield Compared to SCK (kg ha^{-1})	Increase in Yield Compared to SCK (%)	Revenue (CNY ha^{-1})	Increase in Revenue Compared to SCK (%)
SCK	2.75 ± 0.48a	0.30	3001.50	-	-	7803.90	-
SM300	3.00 ± 0.07a	0.33	3301.65	300.15	10.00	8584.29	10.00
SM500	3.47 ± 1.30a	0.38	3801.00	799.50	26.64	9882.60	26.64
SM800	1.89 ±0.78b	0.21	2101.05	-900.45	-30.00	5462.73	-30.00

Note: The average price of Chinese chives in 2021 was 2.6 CNY kg^{-1} locally. Based on the marketable yield of substrate grown Chinese chives in this experiment.

Supplementary Table S6

Supplementary Table S6. Effect of MeJA treatments on marketable yield of hydroponic Chinese chive.

Trentments	Fresh Weight (g $plant^{-1}$)	Marketable Yield (kg/m^2)	Marketable Yield (kg ha^{-1})	Increase in Yield Compared to SCK (kg ha^{-1})	Increase in Yield Compared to SCK (%)	Revenue (CNY ha^{-1})	Increase in Revenue Compared to SCK (%)
HCK	2.43 ± 0.96b	0.44	4402.20	-	-	11445.72	-
HM300	2.47 ± 0.22b	0.46	4602.30	200.10	4.55	11965.98	4.55
HM500	3.30 ± 0.34a	0.60	6003.00	1600.80	36.36	15607.80	36.36
HM800	2.71 ± 0.10ab	0.50	5002.50	600.30	13.64	13006.50	13.64

Note: The average price of Chinese chives in 2021 was 2.6 CNY kg^{-1} locally. Based on the marketable yield of hydroponic Chinese chives in this experiment.

Supplementary Figure S6

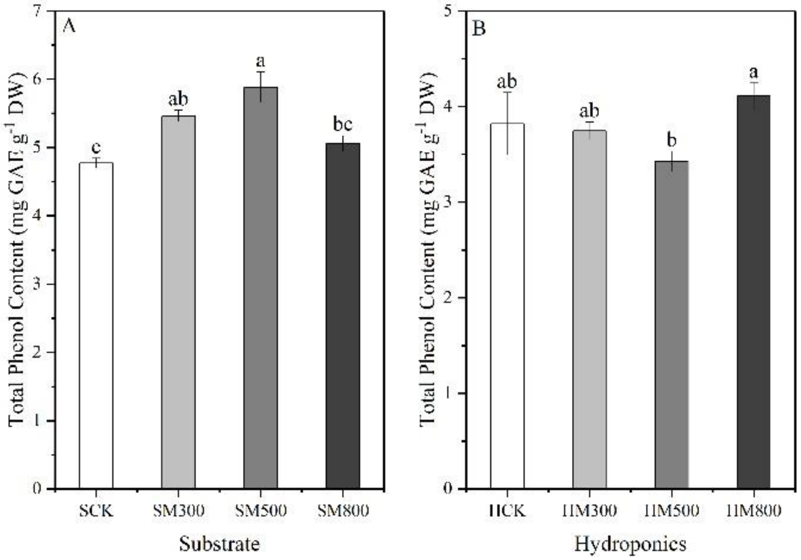

Supplementary Figure S6. Effect of MeJA treatments on total phenol content of substrate-grown (A) and hydroponic (B) Chinese chive.
